# Effects of Enteric Environmental Modification by Coffee Components on Neurodegeneration in Rotenone-Treated Mice

**DOI:** 10.3390/cells8030221

**Published:** 2019-03-07

**Authors:** Ikuko Miyazaki, Nami Isooka, Kouichi Wada, Ryo Kikuoka, Yoshihisa Kitamura, Masato Asanuma

**Affiliations:** 1 Department of Medical Neurobiology, Okayama University Graduate School of Medicine, Dentistry and Pharmaceutical Sciences, Okayama 700-8558, Japan; pi3s34bh@s.okayama-u.ac.jp (N.I.); pjv46mxi@s.okayama-u.ac.jp (K.W.); p32m0bvx@s.okayama-u.ac.jp (R.K.); asachan@cc.okayama-u.ac.jp (M.A.); 2 Department of Clinical Pharmacy, Okayama University Graduate School of Medicine, Dentistry and Pharmaceutical Sciences, Okayama 700-8558, Japan; kitamu-y@cc.okayama-u.ac.jp

**Keywords:** caffeic acid, chlorogenic acid, rotenone, Parkinson’s disease, neuroprotection, dopaminergic neuron, myenteric plexus, enteric glial cell, metallothionein

## Abstract

Epidemiological studies have shown that coffee consumption decreases the risk of Parkinson’s disease (PD). Caffeic acid (CA) and chlorogenic acid (CGA) are coffee components that have antioxidative properties. Rotenone, a mitochondrial complex I inhibitor, has been used to develop parkinsonian models, because the toxin induces PD-like pathology. Here, we examined the neuroprotective effects of CA and CGA against the rotenone-induced degeneration of central dopaminergic and peripheral enteric neurons. Male C57BL/6J mice were chronically administered rotenone (2.5 mg/kg/day), subcutaneously for four weeks. The animals were orally administered CA or CGA daily for 1 week before rotenone exposure and during the four weeks of rotenone treatment. Administrations of CA or CGA prevented rotenone-induced neurodegeneration of both nigral dopaminergic and intestinal enteric neurons. CA and CGA upregulated the antioxidative molecules, metallothionein (MT)-1,2, in striatal astrocytes of rotenone-injected mice. Primary cultured mesencephalic or enteric cells were pretreated with CA or CGA for 24 h, and then further co-treated with a low dose of rotenone (1–5 nM) for 48 h. The neuroprotective effects and MT upregulation induced by CA and CGA in vivo were reproduced in cultured cells. Our data indicated that intake of coffee components, CA and CGA, enhanced the antioxidative properties of glial cells and prevents rotenone-induced neurodegeneration in both the brain and myenteric plexus.

## 1. Introduction

Parkinson’s disease (PD) is a progressive neurodegenerative disease with motor symptoms, such as tremor, akinesia/bradykinesia, rigidity, and postural instability, due to a loss of nigrostriatal dopaminergic neurons, and non-motor symptoms, such as orthostatic hypotension and constipation, caused by peripheral neurodegeneration. Gastrointestinal dysfunction is a particularly prominent non-motor symptom of PD. Several studies have reported that constipation appears approximately 10 to 20 years prior to the presentation of motor symptoms [1,2,3]. Recently, a large-scale prospective study demonstrated that lower bowel movement frequencies predicted the future PD crisis [4]. Braak et al. reported that PD pathology, Lewy bodies and Lewy neuritis, within the central nervous system (CNS), appeared first in the dorsal motor nucleus of vagus, and then extended upward through the brain stem to reach the substantia nigra, eventually leading to motor dysfunction [5]. In addition, several reports have demonstrated that PD pathology is also detected within the enteric nervous system (ENS) [6,7,8]. Therefore, it has been hypothesized that PD pathology spreads from the ENS to the CNS via the vagal nerve [9].

The cause of sporadic PD remains unknown, but both genetic and environmental factors are thought to contribute to PD pathogenesis. Epidemiological studies suggest that pesticide exposure, particularly rotenone and paraquat, increases the risk of PD [10]. Rotenone, a mitochondrial complex I inhibitor, is used to develop animal models of PD, because the toxin induces dopaminergic neuronal loss and PD motor symptoms [11,12]. Studies have demonstrated neurotoxic effects of rotenone in vitro and in vivo [11,13,14]. In addition, rotenone has been shown to reproduce PD pathology in both the CNS and ENS [15,16,17].

Epidemiological studies indicate that coffee consumption reduces the risk of PD to 40–50% [18,19]. Caffeic acid (CA) and chlorogenic acid (CGA), an ester formed between CA and quinic acid, are components of coffee. Various studies have reported that CA and CGA possess antioxidative properties, anti-inflammatory activity, and inhibitory effects on mitochondrial damage. Moreover, these coffee components exert neuroprotective effects against dopaminergic neurotoxicity [20,21,22,23,24,25,26]. However, it is still unknown whether coffee intake provides neuroprotective effects against enteric neuronal damage. The present study explored whether daily oral administrations of CA or CGA could prevent degeneration of central dopaminergic and peripheral enteric neurons in rotenone-treated mice. We established a novel rotenone-treated mouse model that exhibited neurodegeneration in both the CNS and ENS after chronic exposure to a low dose of rotenone (2.5 mg/kg/day) for four weeks. In addition, we examined the expression of the antioxidative molecules metallothionein (MT)-1,2, which are expressed mainly in astrocytes and secreted to the extracellular space, in rotenone-treated mice. Furthermore, we examined the neuroprotective effects of CA and CGA against rotenone-induced neurotoxicity in primary cultured cells from the mesencephalon and intestine.

## 2. Materials and Methods

### 2.1. Animals

All experimental procedures were performed in accordance with the Guideline for Animal Experiments of Okayama University Advanced Science Research Center, and were approved by the Animal Care and Use Committee of Okayama University Advanced Science Research Center. Male C57BL/6J mice at seven weeks of age and pregnant Sprague-Dawley (SD) rats at gestation day 13 were purchased from Charles River Japan Inc. (Yokohama, Japan). C57BL/6J mice and pregnant SD rats were housed with a 12-h light/dark cycle at a constant temperature (23 °C) and given ad libitum access to food.

### 2.2. Rotenone-Injected Mice and Treatment with CA or CGA

In our previous studies, we reported that chronic injection with rotenone (50 mg/kg/day) induced neurodegeneration in the substantia nigra pars compacta (SNpc) and intestinal myenteric plexus in mice [27]. In the current study, to examine the effects of CA and CGA on neurodegeneration in low-dose rotenone-treated mice, male C57BL/6J mice (nine weeks old; approximately 25 g) were subcutaneously injected with rotenone (2.5 mg/kg/day, Sigma-Aldrich, St. Louis, MO, USA) for four weeks using an osmotic mini pump (Alzet, #2004; Durect Corporation, Cupertino, CA, USA). The Alzet osmotic pump was filled with rotenone (10.4 mg/mL) dissolved in the vehicle solution, consisting of equal volumes of dimethylsulphoxide (DMSO) and polyethylenglycol (PEG). Mice were anesthetized by isoflurane inhalation. Rotenone-filled pumps were implanted under the skin on the backs of mice. Control mice received the vehicle solution.

**Figure 1:**
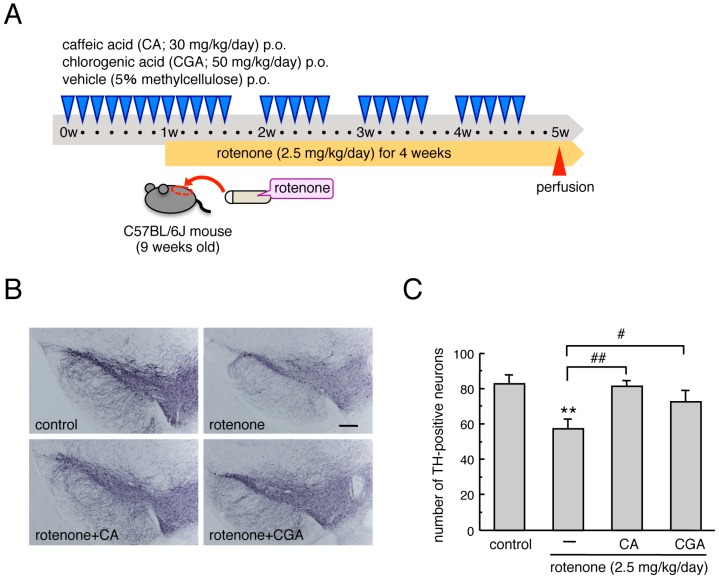
Administrations of CA or CGA prevented degeneration of dopaminergic neurons in the SNpc of rotenone-treated mice. (**A**) Schematic illustration of the experimental protocol. Male C57BL/6J mice were injected subcutaneously with rotenone (2.5 mg/kg/day) for four weeks using an osmotic mini pump. Mice were orally administered CA (30 mg/kg/day) or CGA (50 mg/kg/day), dissolved in 5% methylcellulose, daily for one week before rotenone exposure, and then 5 days/week during the four weeks of rotenone treatment. (**B**) Representative photomicrographs of immunohistochemistry for TH in the SNpc of rotenone-treated mice after treatment with CA or CGA. Scale bar = 200 µm. (**C**) Changes in the number of TH-positive nigral neurons after administration of CA or CGA. Each value is the mean ± SEM (n = 6–7). ** *p* < 0.01 vs. the vehicle-treated control group, ^#^ *p* < 0.05, ^##^ *p* < 0.01 between the two indicated groups.

Mice were orally administered CA (30 mg/kg/day) or CGA (50 mg/kg/day) dissolved in 5% methylcellulose daily for one week before rotenone exposure, and then 5 days/week during the four weeks of rotenone treatment (Figure 1A). The dosages of CA and CGA were determined based on previous reports [22,24,25]. One day after the rotenone treatment period, mice were perfused transcardially with a 4% paraformaldehyde (PFA) fixative for immunohistochemical analysis.

### 2.3. Cell Culture of Mesencephalic Neurons and Astrocytes

Primary cultured mesencephalic neurons and astrocytes were prepared from the mesencephalon of SD rat embryos at 15 days of gestation [28]. Neuronal and astrocyte co-cultures were constructed by directly seeding astrocytes onto neuronal cell cultures. To prepare enriched neuronal cultures, the mesencephalon was dissected, cut into small pieces with scissors, and then incubated for 15 min in 0.125% trypsin-EDTA at 37 °C. After centrifugation (1500× *g*, 3 min), the resulting cell pellet was treated with a 0.004% DNase I solution, containing 0.003% trypsin inhibitor, for 7 min at 37 °C. Following centrifugation (1500× *g*, 3 min), the cell pellet was gently resuspended in a small volume of Dulbecco’s modified Eagle’s medium (DMEM) with 4.5 g/L d-glucose (Invitrogen, San Diego, CA, USA), 10% fetal bovine serum (FBS), 4 mM L-glutamine, and 60 mg/L kanamycin sulfate (growth medium; DMEM–FBS). Resuspended cells were plated in the same medium at a density of 2 × 10^5^ cells/cm^2^ in four-chamber culture slides coated with poly-d-lysine (Falcon, Corning, NY, USA). Within 24 h of the initial plating, the medium was replaced with fresh DMEM-FBS medium supplemented with 2 µM cytosine-β-d-arabinofuranoside (Ara-C) to inhibit glial cell replication. Cells were incubated in this medium for three days. To obtain mesencephalic astrocytes, small pieces of mesencephalon were treated with 0.125% trypsin followed by 0.004% DNase I, as described above. Cells were plated at a density of 2 × 10^5^ cells/cm^2^ in poly-d-lysine-coated 6-well plates (Falcon) in DMEM-FBS medium. After incubation for seven days, cells were subcultured, and then seeded, at a density of 4 × 10^4^ cells/cm^2^, directly onto mesencephalic neuronal cell layers that had been cultured in four-chamber culture slides for 4 days. The co-cultures were incubated for a further two days before beginning any treatment. To prepare astrocyte cultures, cells were subcultured as described above, plated in DMEM-FBS medium at a density of 2 × 10^4^ cells/cm^2^ in poly-d-lysine-coated four-chamber culture slides, and then incubated for one week. All cultures were maintained at 37 °C in a 5%/95% CO_2_/air mixture.

### 2.4. Cell Culture of Enteric Neurons and Glial Cells

Enteric neuronal and glial co-cultures were prepared from the intestine of SD rat embryos at 15 days of gestation [29]. Intestines were dissected and kept on ice in Hank’s buffered salt solution (HBSS, Sigma-Aldrich) supplemented with 50 µg/mL streptomycin and 50 U/mL penicillin (Invitrogen). After washing with fresh HBSS, intestines were cut into small pieces with scissors in DMEM/F12 (1:1) medium (Invitrogen) with 50 µg/mL streptomycin, 50 U/mL penicillin (DMEM/F12 medium), and 10% FBS, and then centrifuged (1500× *g*, 3 min). The resulting cell pellet was treated with 0.125% trypsin (Invitrogen) for 15 min at 37 °C. After centrifugation (1500× *g*, 10 min), the resulting cells were treated with 0.01% (*v*/*v*) DNase I (Sigma-Aldrich) for 10 min at 37 °C. After centrifugation (100× *g*, 10 min), the cell pellet was gently re-suspended in a small volume of DMEM/F12 medium containing 10% FBS, and then plated in the same medium at a density of 5 × 10^4^ cells/cm^2^ in four-chamber culture slides previously coated for 6 h with a solution of 0.5% (*v*/*v*) gelatin (Sigma-Aldrich). Within 24 h of the initial plating, the medium was replaced with fresh DMEM/F12 medium without FBS but containing 1% N-2 and 1% G-5 supplements (Invitrogen). Half of the medium was replaced every two days, and cell cultures were maintained for 13 days at 37 °C in a 5%/95% CO_2_/air mixture.

### 2.5. Cell Treatments

Fresh solution of rotenone in DMSO were prepared before each experiment and then diluted to their final concentrations in the appropriate growth medium (final concentration of DMSO: 0.005% *v*/*v*). To examine the effects of CA and CGA on rotenone-induced dopaminergic neurotoxicity, mesencephalic neuronal and astrocyte co-cultures were treated with CA (10 or 25 µM) or CGA (25 µM) in 0.2% DMSO in growth medium for 24 h. The concentrations of CA and CGA were determined based on previous reports [30,31,32]. After incubation for 24 h, the medium containing CA or CGA was discarded, and then cells were treated with CA (10 or 25 µM) or CGA (25 µM) and rotenone (1, 2.5, or 5 nM) for 48 h. To examine the effects of CA and CGA on MT-1,2 expression in mesencephalic astrocytes, astrocyte cultures were treated with CA (10 or 25 µM) or CGA (25 µM) for 24 h in advance, and then treated with CA (10 or 25 µM) or CGA (25 µM), with or without rotenone (1, 2.5, or 5 nM) for a further 48 h.

To determine the effects of CA and CGA on rotenone-induced enteric neuronal loss and glial MT-1,2 expression, enteric neuronal and glial co-cultures were treated with CA (10 or 25 µM) or CGA (25 µM) in DMEM/F12 medium containing 0.2% DMSO and 1% N-2 and 1% G-5 supplements for 24 h, followed by treatment with CA (10 or 25 µM) or CGA (25 µM) and rotenone (1, 2.5, or 5 nM) for 48 h.

### 2.6. Immunohistochemistry

To prepare slices of the brain and intestine, mice were perfused with ice-cold saline followed by 4% PFA under deep pentobarbital anesthesia (70 mg/kg, i.p.). The perfused brains and intestines were removed immediately and post-fixed for 24 h or 2 h in 4% PFA, respectively. Following cryoprotection in 15% sucrose in phosphate buffer (PB) for 48 h, the brains and intestines were snap-frozen with powdered dry ice and 20-µm-thick coronal or transverse sections were cut on a cryostat. Brain slices were collected at levels containing the mid-striatum (+0.6 to +1.0 mm from the bregma) and the SNpc (−2.8 to −3.0 mm from bregma). For immunostaining of tyrosine hydroxylase (TH) in the SNpc, brain slices were treated with 0.5% H_2_O_2_ for 30 min at room temperature (RT), blocked with 1% normal goat serum for 30 min, and incubated for 18 h at 4 °C with a rabbit anti-TH antibody (1:1000; Millipore, Temecula, CA, USA) diluted in 10 mM phosphate-buffered saline (PBS) containing 0.2% Triton X-100 (0.2% PBST). After washing in 0.2% PBST (3 × 10 min), slices were reacted with biotinylated goat anti-rabbit IgG secondary antibody for 2 h at RT. After washing, the sections were incubated with an avidin-biotin peroxidase complex for 1 h at RT. TH-immunopositive signals were visualized by DAB, nickel, and H_2_O_2_. To examine effects of CA and CGA treatment on astrocytic MT-1,2 expression in rotenone-injected mice, the striatal sections were incubated in 1% normal goat serum for 30 min at RT, and then reacted with mouse anti-MT-1,2 (1:100; Dako Cytomation, Glostrup, Denmark) or rabbit anti-S100β (1:5000; Dako Cytomation) antibodies for 18 h at 4 °C. After washing, slices were reacted with Alexa Fluor 594-conjugated goat anti-mouse IgG or Alexa Fluor 488-conjugated goat anti-rabbit IgG secondary antibodies (1:1000; Invitrogen) for 2 h at RT. To visualize the myenteric plexus or enteric glial cells in the intestine, intestinal sections were incubated in 1% normal goat serum for 30 min at RT, and then reacted with rabbit anti-β-tubulin III (1:100; GeneTex, Inc., Irvine, CA, USA) or rabbit anti-GFAP (1:10,000; Novus Biologicals, Centennial, CO, USA) antibody, respectively, for 18 h at 4 °C. After washing, slices were reacted with Alexa Fluor 488-conjugated goat anti-rabbit IgG secondary antibody (1:1000; Invitrogen) for 2 h at RT. The striatal and intestinal slices were then counterstained with Hoechst 33342 nuclear stain (10 µg/mL) for 2 min. Cells cultured on chamber slides were fixed with 4% PFA for 30 min at RT, blocked with 2.5% normal goat serum for 20 min, and then reacted for 18 h at 4 °C with the following primary antibodies diluted in 0.1% PBST: rabbit anti-TH (1:1000; Millipore); mouse anti-MT-1,2 (1:100; DAKO Cytomation); rabbit anti-GFAP (1:2000; Dako Cytomation); or mouse anti-β-tubulin III (1:10,000; Sigma-Aldrich). After washing in 10 mM PBS, pH 7.4 (3 × 10 min), cells were reacted with Alexa Fluor 488-conjugated goat anti-rabbit IgG or Alexa Fluor 594-conjugated goat anti-mouse IgG secondary antibodies (1:500; Invitrogen) for 1.5 h at RT. Finally, cells were counterstained with Hoechst 33342 nuclear stain (10 µg/mL) for 2 min and washed prior to mounting with Fluoromounting medium (Dako Cytomation).

All slides were analyzed under a fluorescence microscope (BX50-FLA or BX53; Olympus Tokyo, Japan) and cellSens imaging software (Olympus), using a mercury lamp (USHIO INC., Tokyo, Japan) through 360–370 nm, 470–495 nm, or 530–550 nm band-pass filters to excite Hoechst 33342, Alexa Fluor 488, or Alexa Fluor 594, respectively. Light emission from Hoechst 33342, Alexa Fluor 488, or Alexa Fluor 594 was collected through a 420 nm long-pass filter, a 510–550 nm band-pass filter, or a 590 nm long-pass filter, respectively. Localization of β-tubulin III- and GFAP signals was confirmed by confocal laser-scanning microscopy (LSM 780; Zeiss, Oberkochen, Germany). Light emitted from Hoechst 33342, Alexa Fluor 488, or Alexa Fluor 594 was collected through a 420–470 nm band-pass filter, a 500–550 nm band-pass filter, or a 570–640 nm band-pass filter, respectively. Images were taken at a magnification of 400× and recorded using the Windows-based LSM program (ZEN lite 2012 64bit version, Zeiss). Adobe Photoshop CS4 software (v11.0) was used for digital amplification of the images.

### 2.7. Quantification Procedures

The number of TH-immunopositive neurons in the SNpc was counted manually under a microscope at 100× magnification. The boundary between the SNpc and ventral tegmental area was defined by a line extending dorsally from the most medial boundary of the cerebral peduncle. The numbers of MT-1,2- and S100β-immunopositive cells in the dorsal striatum of rotenone-treated mice were counted manually using a microscope at a magnification of 400×. The number of MT- or S100β-positive cells and the ratio of MT-positive cells to S100β-positive cells were evaluated in each section. The immunoreactivity of β-tubulin III or GFAP in the myenteric plexus of the intestine was analyzed under 400× magnification and quantified using cellSens imaging software (v1.16, Olympus). The integrated density of each signal was calculated as follows: integrated density = (signal density in the myenteric plexus-background density) × area of positive signal in the plexus.

TH-immunopositive cells in mesencephalic neuronal and astrocyte co-cultures were counted under a microscope in all areas of each chamber slide. Cell viability data are presented as a percentage of the control. The number of MT-1,2-immunopositive cells in mesencephalic astrocyte cultures was counted in 8–10 randomly chosen fields in a chamber under 200× magnification, and expressed as the percentage of MT-1,2-immunopositive astrocytes among the total cell population. The signal intensity of β-tubulin III and MT-1,2 in enteric neuronal and glial co-cultures was analyzed in 3–6 randomly chosen fields in a chamber under 200× magnification and quantified using cellSens imaging software.

### 2.8. Statistical Analyses

All statistical analyses were performed using KaleidaGraph v4.0 software. Data are presented as means ± SEM. Comparisons between multiple groups were performed using a one-way ANOVA followed by a *post hoc* Fisher’s least significant difference test. A *p*-value < 0.05 was considered statistically significant.

## 3. Results

### 3.1. Administrations of CA or CGA Prevented Dopaminergic Neurodegeneration in Rotenone-Treated Mice

Chronic subcutaneous treatment with a low dose of rotenone (2.5 mg/kg/day) significantly decreased the number of TH-positive dopaminergic neurons in the SNpc. Repeated oral administration of CA (30 mg/kg) or CGA (50 mg/kg) ameliorated the reduction of nigral TH-positive cells in rotenone-treated mice (Figure 1B,C).

### 3.2. Administrations of CA or CGA Increased MT-1,2 Expression in Astrocytes in the Striatum of Rotenone-Treated Mice

**Figure 2:**
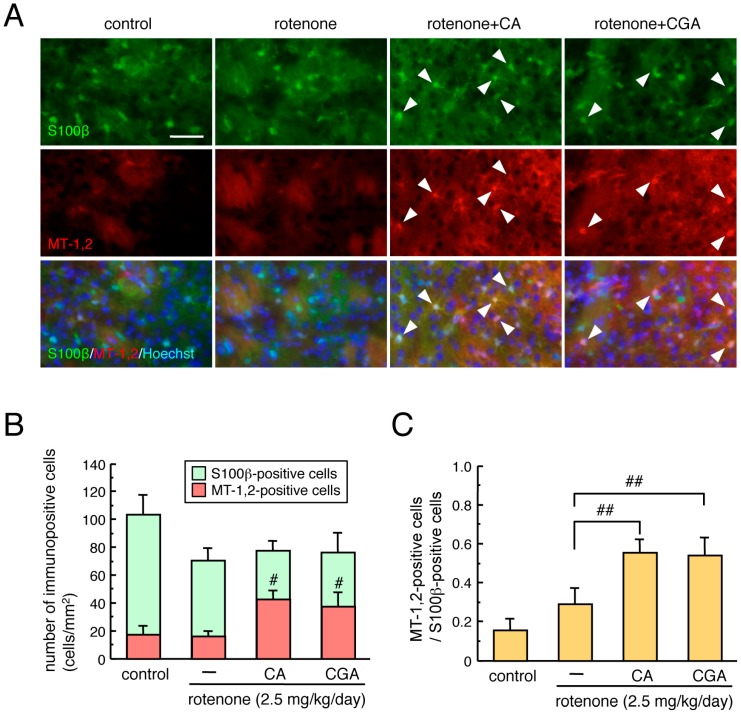
Effects of CA or CGA administrations on astrocytic MT-1,2 expression in the striatum of rotenone-treated mice. (**A**) Representative photomicrographs of MT-1,2 and S100β double immunostaining in the striatum of rotenone (2.5 mg/kg/day)-treated mice after treatment with CA (30 mg/kg/day) or CGA (50 mg/kg/day). Green: S100β-positive astrocytes. Red: MT-1,2-positive cells. Blue: nuclear staining with Hoechst 33342. Solid arrowheads: MT-1,2-positive astrocytes. Scale bar = 50 µm. (**B**,**C**) Quantitation of MT-1,2 and S100β expression in the striatum of rotenone-treated mice after treatment with CA or CGA. (**B**) Number of immunopositive cells, (**C**) proportion of MT-1,2-positive cells/S100β-positive cells. Data are means ± SEM (n = 6–7). ^#^ *p* < 0.05, ^##^ *p* < 0.01 vs. the rotenone-treated group.

To examine the effects of CA and CGA treatment on antioxidative molecules in astrocytes of rotenone-treated mice, we performed double immunostaining of the astrocyte marker S100β and MT-1,2 in striatal brain slices. We used an anti-S100β, but not an anti-GFAP, antibody to visualize astrocytes in the striatum. Since the anti-GFAP antibody detected mainly fibrous activated astrocytes, it was difficult to assess MT-1,2 expression in all types of astrocytes, including protoplasmic astrocytes. Therefore, we chose an anti-S100β antibody to detect striatal astrocytes. A nonsignificant trend toward decreased numbers of S100β-positive astrocytes were seen after rotenone treatment. Administration of CA or CGA significantly increased the number of MT-positive astrocytes in the striatum of mice (Figure 2A,B). The MT-positive/S100β-positive cell ratio was significantly increased by either CA or CGA treatment (Figure 2C).

### 3.3. Administration of CA or CGA Prevented Neurodegeneration in the Intestinal Myenteric Plexus of Rotenone-Treated Mice

**Figure 3:**
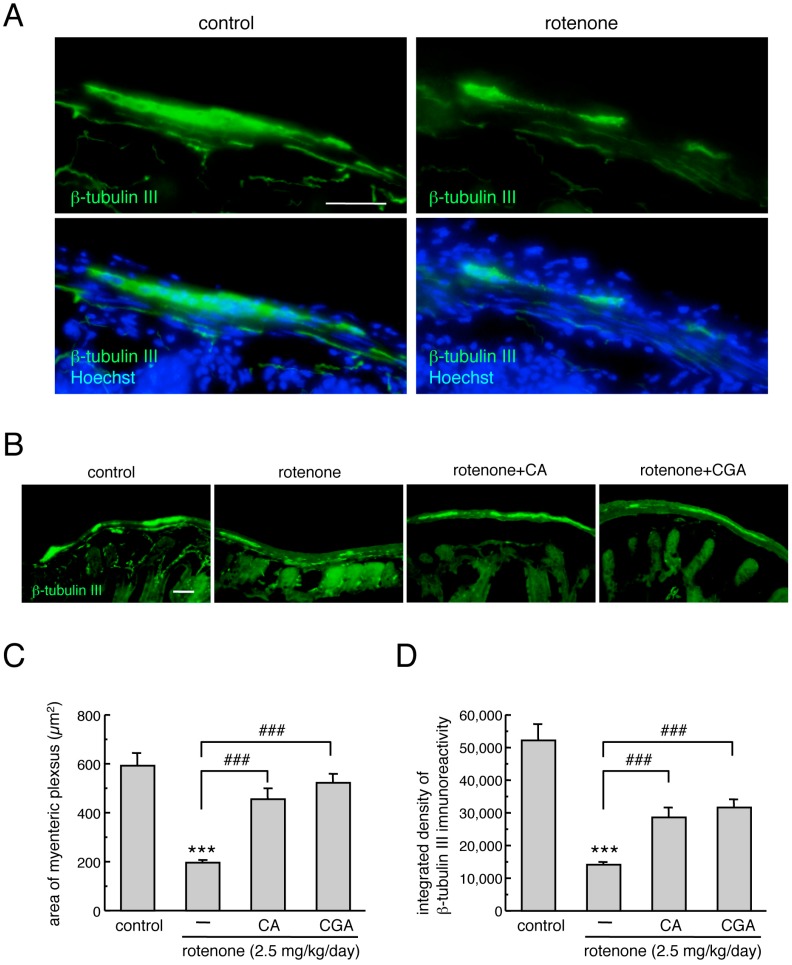
Administrations of CA or CGA prevented the degeneration of enteric neurons in the intestinal myenteric plexus of rotenone-treated mice. (**A**) Representative photomicrographs of immunohistochemistry for β-tubulin III in the intestine of mice. Green: β-tubulin III-positive neurons. Blue: nuclear staining with Hoechst 33342. Scale bar = 50 µm. (**B**) Representative photomicrographs of β-tubulin III-positive neurons in the intestine of rotenone-treated mice after treatment with CA (30 mg/kg/day) or CGA (50 mg/kg/day). Scale bar = 50 µm. (**C**,**D**) Quantitation of β-tubulin III-positive signals in the intestine. (**C**) Area of β-tubulin III-positive myenteric plexus, (**D**) integrated density of β-tubulin III immunoreactivity. Data are means ± SEM (n = 6–7). *** *p* < 0.001 vs. the vehicle-treated control group, ^###^ *p* < 0.001 between the two indicated groups.

To examine the neuroprotective effects of CA and CGA on the myenteric plexus in the small intestine of rotenone-treated mice, we performed immunostaining of the neuronal marker, β-tubulin III. To confirm the distribution of the myenteric plexus in the intestine, nuclear staining was performed using Hoechst 33342. Apparent β-tubulin III-positive signals were detected in the intestinal myenteric plexus of mice (Figure 3A). Chronic subcutaneous treatment with low-dose rotenone for four weeks significantly decreased the area of β-tubulin III-positive myenteric plexus (Figure 3A–C) and β-tubulin III immunoreactivity (Figure 3A,B,D) in the intestine. Repeated administration of CA or CGA significantly prevented this reduction in β-tubulin III-positive signals in the myenteric plexus of rotenone-treated mice (Figure 3B–D).

### 3.4. Administration of CA or CGA Had No Effect on Enteric Glial Cells in Rotenone-Treated Mice

**Figure 4:**
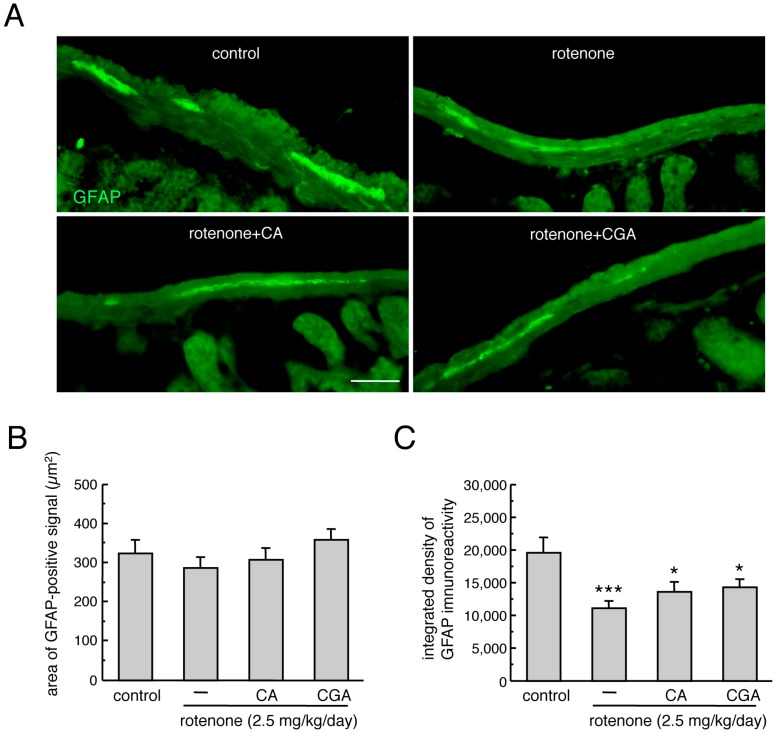
Effects of CA or CGA administrations on enteric glial cells in the intestines of rotenone-treated mice. (**A**) Representative photomicrographs of immunohistochemistry for GFAP in the intestines of rotenone-treated mice after treatment with CA (30 mg/kg/day) or CGA (50 mg/kg/day). Scale bar = 50 µm. (**B**,**C**) Quantitation of GFAP-positive signals in the intestine of mice. (**B**) Area of GFAP-positive signal, (**C**) integrated density of GFAP immunoreactivity. Data are means ± SEM (n = 6–7). * *p* < 0.05, *** *p* < 0.001 vs. the vehicle-treated control group.

To examine the effects of CA and CGA on enteric glial cells in the small intestine of rotenone-treated mice, we performed immunostaining of the glial marker, GFAP [33]. Chronic subcutaneous treatment with a low dose of rotenone for four weeks had no effect on the area of GFAP-positive signal (Figure 4A,B), but significantly decreased GFAP immunoreactivity (Figure 4A,C) in the intestine. Repeated administration of CA or CGA did not prevent this reduction in GFAP-positive signal in the intestine of rotenone-treated mice (Figure 4A–C).

### 3.5. Treatment with CA or CGA Inhibited Rotenone-Induced Dopaminergic Neuronal Loss in Mesencephalic Neuronal and Astrocyte Co-Cultures

**Figure 5:**
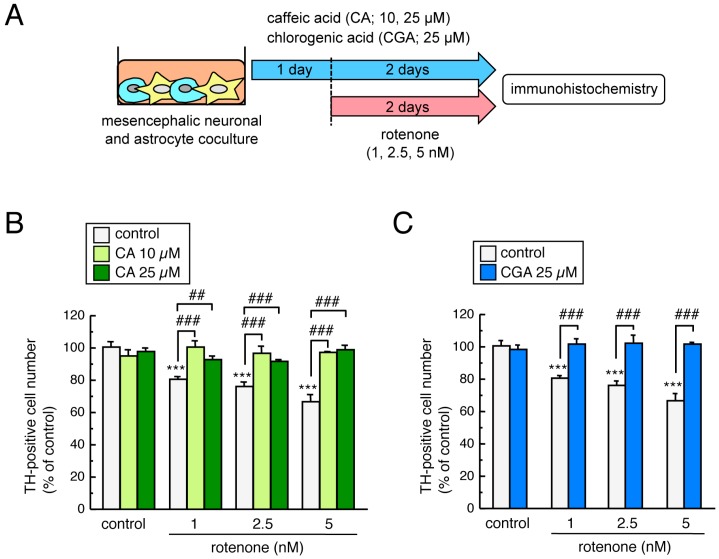
Neuroprotective effects of CA and CGA against rotenone-induced dopaminergic neurotoxicity in cultured mesencephalic cells. (**A**) Schematic illustration of the experimental protocol. Mesencephalic neuronal and astrocyte co-cultures were pretreated with CA (10 or 25 µM) or CGA (25 µM) for 24 h and co-treated with rotenone (1–5 nM) for a further 48 h. (**B**,**C**) Changes in the number of TH-positive neurons after treatment with rotenone and CA (**B**) or CGA (**C**). Data are means ± SEM (n = 4). *** *p* < 0.001 vs. each control group, ^##^ *p* < 0.01, ^###^ *p* < 0.001 between the two indicated groups.

To examine the neuroprotective effects of CA and CGA on rotenone-induced dopaminergic neurodegeneration in cultured cells, mesencephalic neuronal and astrocyte co-cultures were pretreated with CA (10 or 25 µM) or CGA (25 µM) for 24 h and co-treated with low-dose rotenone (1–5 nM) for a further 48 h (Figure 5A). Exposure to a low dose of rotenone significantly decreased the number of TH-positive dopaminergic neurons. Both CA and CGA treatment significantly and completely inhibited this reduction in the number of TH-positive cells (Figure 5B,C).

### 3.6. Treatment with CA or CGA Upregulated MT-1,2 in Mesencephalic Astrocytes

**Figure 6:**
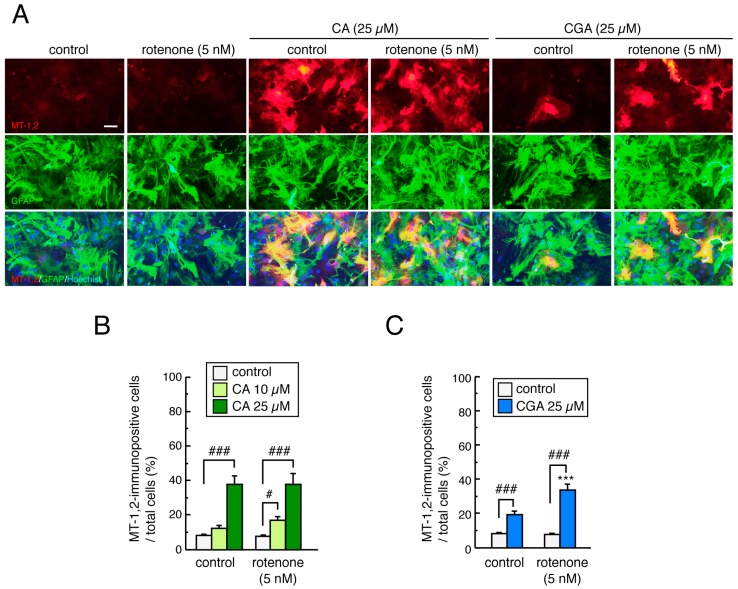
Effects of treatment with CA or CGA followed by rotenone exposure on MT-1,2 expression in mesencephalic astrocyte cultures. Astrocyte cultures were pretreated with CA (10 or 25 µM) or CGA (25 µM) for 24 h and co-treated with rotenone (1–5 nM) for a further 48 h. (**A**) Representative photomicrographs of MT-1,2 and GFAP double immunostaining in astrocyte cultures. Red: MT-1,2-positive signals. Green: GFAP-positive astrocytes. Blue: nuclear staining with Hoechst 33342. Scale bar = 50 µm. (**B**,**C**) Quantitation of MT-1,2-positive signals in astrocyte cultures after treatment with rotenone and CA (**B**) or CGA (**C**). Each value is the mean ± SEM (n = 8–13) expressed as the percentage of the MT-1,2-immunopositive astrocytes in the total cell population. *** *p* < 0.001 vs. each control group, ^#^ *p* < 0.05, ^###^ *p* < 0.001 between the two indicated groups.

To examine the effects of treatment with CA or CGA, with or without rotenone, on MT-1,2 expression in mesencephalic astrocytes, astrocyte cultures were pretreated with CA (10 or 25 µM) or CGA (25 µM) for 24 h and co-treated with rotenone (1–5 nM) for 48 h. Rotenone had no effect on MT-1,2 expression in astrocytes even at the highest dose (5 nM). Treatment with CA or CGA significantly increased MT-1,2 expression in astrocytes even after rotenone exposure (Figure 6A–C). Interestingly, the induction of MT-1,2 by CGA (25 µM) was higher in the rotenone-treated astrocytes than in the untreated control group (Figure 6C).

### 3.7. Treatment with CA or CGA Inhibited Rotenone-Induced Enteric Neuronal Loss in Enteric Neuronal and Glial Co-Cultures

**Figure 7:**
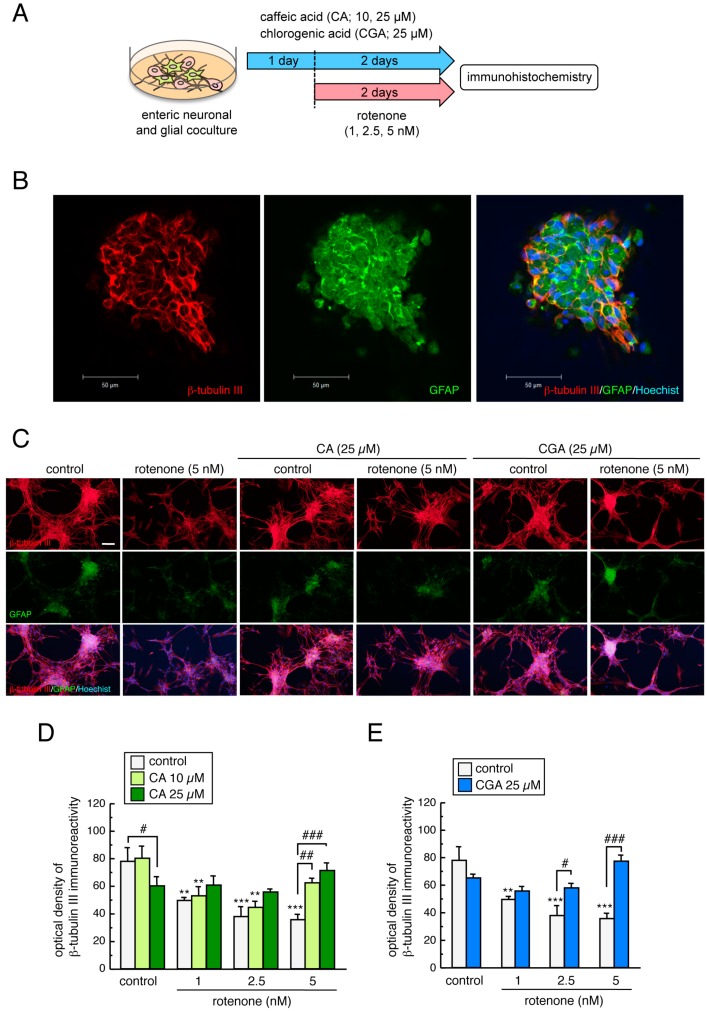
Neuroprotective effects of CA and CGA against rotenone-induced enteric neurotoxicity in primary cultured intestinal cells. (**A**) Schematic illustration of the experimental protocol. Enteric neuronal and glial co-cultures were pretreated with CA (10 or 25 µM) or CGA (25 µ) for 24 h and co-treated with rotenone (1–5 nM) for further 48 h. (**B**) Localization of β-tubulin III- and GFAP-positive signals. Red: β-tubulin III-positive enteric neuronal cells. Green: GFAP-positive enteric glial cells. Blue: nuclear staining with Hoechst 33342. Scale bar = 50 µm. (**C**) Representative photomicrographs of immunohistochemistry for β-tubulin III and GFAP in enteric cell cultures after treatment with rotenone (5 nM) and CA (25 µM) or CGA (25 µM). Red: β-tubulin III-positive enteric neuronal cells. Green: GFAP-positive enteric glial cells. Blue: nuclear staining with Hoechst 33342. Scale bar = 100 µm. (**D**,**E**) Quantitation of β-tubulin III-positive signals in enteric cell cultures after treatment with rotenone and CA (**D**) or CGA (**E**). Data are means ± SEM (n = 3–6). ** *p* < 0.01, *** *p* < 0.001 vs. each control group; ^#^ *p* < 0.05, ^##^ *p* < 0.01, ^###^ *p* < 0.001 between the two indicated groups.

To examine whether low-dose rotenone exposure induced enteric neurotoxicity, and whether CA or CGA treatment could prevent these effects, we prepared enteric neuronal and glial co-cultures from the intestines of SD rat embryos. Enteric neuronal and glial co-cultures were pretreated with CA or CGA for 24 h, and then co-treated with a low dose of rotenone (1–5 nM) for a further 48 h (Figure 7A). We successfully detected β-tubulin III-positive enteric neuronal cells and GFAP-positive glial cells. Enteric glial were seen in the nerve plexus (Figure 7B). Exposure to a low dose of rotenone (1–5 nM) for 48 h significantly decreased β-tubulin III immunoreactivity in cultured enteric cells. Treatment with CA (10, 25 µM) or CGA (25 µM) significantly ameliorated the reduction in β-tubulin III-positive signals induced by rotenone exposure (Figure 7C–E). These results indicate that both CA and CGA provide neuroprotection against rotenone-induced enteric neurotoxicity. Interestingly, CA and CGA could both protect enteric neurons against higher doses of rotenone exposure (5 nM), but not against lower doses (1 or 2.5 nM, Figure 7D,E).

### 3.8. Treatment with CA or CGA Inhibited Rotenone-Induced MT-1,2 Reduction in Enteric Glial Cells

**Figure 8:**
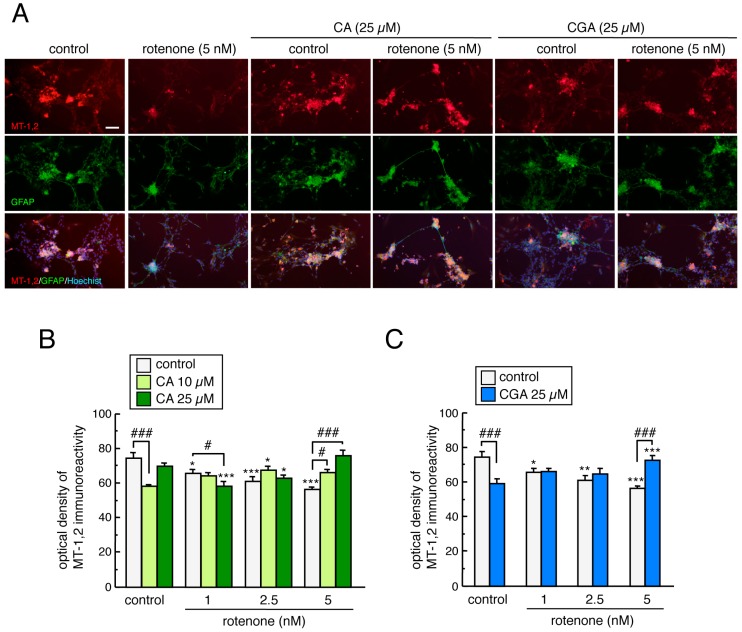
Treatment with CA or CGA inhibits rotenone-induced MT downregulation in the enteric glial cells. Enteric neuronal and glial co-cultures were pretreated with CA (10 or 25 µM) or CGA (25 µM) for 24 h and co-treated with rotenone (1–5 nM) for a further 48 h. (**A**) Representative photomicrographs of immunohistochemistry for MT-1,2 and GFAP in enteric cell cultures after treatment with rotenone (5 nM) and CA (25 µM) or CGA (25 µM). Red: MT-1,2-positive signals. Green: GFAP-positive enteric glial cells. Blue: nuclear staining with Hoechst 33342. Scale bar = 100 µm. (**B**,**C**) Quantitation of MT-1,2-positive signals in the enteric cell cultures after treatment with rotenone and CA (**B**) or CGA (**C**). Data are means ± SEM (n = 3–8). * *p* < 0.05, ** *p* < 0.01, *** *p* < 0.001 vs. each control group; ^#^ *p* < 0.05, ^###^ *p* < 0.001 between the two indicated groups.

We examined the effects of CA and CGA treatment on MT-1,2 expression in rotenone-treated enteric glial cells by double immunostaining of GFAP and MT-1,2. MT-positive signals were localized to cultured GFAP-positive enteric glial cells (Figure 8A). In contrast to the results from cultured mesencephalic cells, rotenone exposure significantly decreased MT-1,2 expression in enteric glial cells and both CA (10 or 25 µM) and CGA (25 µM) inhibited the rotenone-induced downregulation of MT (Figure 8B,C). Remarkably, these inhibitory effects of CA or CGA were only observed in enteric cells treated with highest dose of rotenone (5 nM) and not in those treated with lower doses (1 or 2.5 nM, Figure 8B,C). This was in agreement with the finding that these coffee components have protective effects against rotenone-induced enteric neurotoxicity (Figure 7D,E).

## 4. Discussion

The present study demonstrated that CA and CGA upregulated MT-1,2 in astrocytes and exerted neuroprotective effects against rotenone-induced dopaminergic neurodegeneration in mesencephalic neuronal and astrocyte co-cultures. In the enteric neuronal and glial co-cultures, rotenone treatment reduced MT-1,2 expression in glial cells and produced enteric neuronal loss, which were prevented by CA or CGA treatment. Furthermore, oral administration of CA or CGA exerted neuroprotective effects against neurodegeneration in the nigral dopaminergic neurons and the enteric neurons in the intestinal myenteric plexus of rotenone-treated mice. In this study, we used a novel mouse model produced by 4-week administration of a low dose of rotenone (2.5 mg/kg/day), which corresponds to the environmental exposure levels of rotenone via pesticides. Chronic subcutaneous injection of rotenone induced neurodegeneration in both the CNS and ENS. Interestingly, rotenone-induced neurotoxicity was more severe in the intestinal myenteric plexus than in the SNpc. Considering the epidemiological data showing an inverse association of daily coffee consumption with PD risk [18,19], we examined the neuroprotective effects of CA and CGA against rotenone neurotoxicity. Animals were treated with these coffee components by daily oral administration for 1 week before rotenone exposure, and then 5 days/week during the four weeks of rotenone treatment. The dosages of CA (30 mg/kg/day) and CGA (50 mg/kg/day) were determined based on previous reports [22,24,25]. It has been reported that one cup of coffee contains 70–350 mg of CA [34] and approximately 250 mg of CGA [35]. In addition, it is known that the daily intake of CA in coffee drinkers is 0.1–1 g [36]. Therefore, the dosages of CA and CGA in the present experiments seem high, since it would be necessary to drink 5–10 cups of coffee per day to achieve their neuroprotective effects in humans.

Treatment with CA or CGA significantly prevented rotenone-induced neurodegeneration of nigral dopaminergic neurons and intestinal enteric neurons. Previous studies have reported that CA and CGA provide dopaminergic neuroprotection in various models of PD [20,21,22,23,24,25,26]. In those reports, CA and CGA showed antioxidative properties [23,24], anti-inflammatory activity [21,24,25,26], and inhibitory effects of mitochondrial damage [20]. Various reports have demonstrated that CA and CGA activate the nuclear factor erythroid-2-related factor 2 (Nrf2) antioxidant pathway [37,38,39,40,41,42]. In this study, we showed that CA or CGA treatment upregulated the antioxidative molecules, MT-1,2, in mesencephalic astrocyte cultures and striatal astrocytes of rotenone-treated mice. MT is a low-molecular weight, cysteine-rich (30% of the protein), inducible protein that binds to metals, such as zinc, copper, and cadmium, and contributes to metal homeostasis and detoxification [43]. In addition, MT directly scavenges free radicals [44,45]. The mammalian MT family comprises four isoforms: MT-1, MT-2, MT-3, and MT-4. The two major isoforms, MT-1 and -2, are often considered physiologically equivalent, because they are expressed in most organs and show coordinated induction in response to various stimulants such as metals, hormones, cytokines, inflammation, and oxidative stress [43,46,47]. We previously reported that MT-1,2 were upregulated specifically in striatal astrocytes by activation of the Nrf2 pathway in response to oxidative stress and they protected nigral dopaminergic neurons [48]. In addition, we have recently discovered that MT-1,2-knockdown in astrocytes aggravates rotenone-induced dopaminergic neurotoxicity. Therefore, in the present study, we focused on MT-1,2 expression in astrocytes as neuroprotective molecules after CA or CGA treatment. Our findings suggest that both CA and CGA could prevent dopaminergic neurodegeneration induced by the upregulation of astrocytic antioxidants in parkinsonian mice. Although the mechanism of MT upregulation by CA and CGA is still unknown, CA- and CGA-induced Nrf2 activation may be involved.

Constipation is the most prominent non-motor symptom in PD, and it might precede motor symptoms by 10–20 years [1,2,3]. Accumulating evidence indicates that the ENS is involved in the pathological progression of PD towards the CNS [15,49]. Therefore, it is desirable to find approaches that can inhibit enteric neurodegeneration. As mentioned above, various reports demonstrated the neuroprotective action of CA and CGA against dopaminergic neurodegeneration in vitro and in vivo [20,21,22,23,24,25,26]. However, few experiments have been performed to explore the neuroprotective effects of these coffee components against enteric neuronal damage. Here, we demonstrated that rotenone treatment induced enteric neuronal degeneration in mice, and that treatment with CA or CGA prevented neuronal loss in the myenteric plexus in these mice. In addition, we explored MT-1,2 expression in cultured enteric cells after treatment with rotenone and coffee components. GFAP-positive enteric glial cells accumulated in the nerve plexus, and MT-1,2 were expressed specifically in enteric glial cells. Furthermore, CA or CGA prevented the rotenone-induced downregulation of MT in cultured cells. These findings suggest that CA and CGA could protect enteric neurons against rotenone toxicity by targeting the antioxidative properties of enteric glial cells. Moreover, we examined MT-1,2 expression in enteric glial cells in rotenone-treated mice, but did not detect obvious MT signals in the intestine. Thus, it is still unclear whether MT is involved in the neuroprotective effects of CA and CGA against rotenone-induced enteric neurotoxicity in vivo. This will require further investigation.

In the present study, we used mesencephalic neuronal and astrocyte co-cultures to examine whether CA and CGA could exert neuroprotective effects against rotenone-induced dopaminergic neurotoxicity. In preliminary experiments, we observed that low-dose rotenone (1–5 nM) did not reduce the number of dopaminergic neurons in the mesencephalic neuronal and striatal co-culture. Therefore, the effects of CA and CGA on MT-1,2 expression were examined in mesencephalic astrocyte cultures, but not striatal astrocyte cultures. In the present study, both CA and CGA significantly increased MT-1,2 expression in mesencephalic astrocyte cultures. Interestingly, treatment with CGA induced MT expression especially in rotenone-exposed astrocytes. In addition, although treatment with CA (10 µM) or CGA (25 µM) reduced MT expression in enteric glial cells, these coffee components prevented the downregulation of MT when used in combination with rotenone (5 nM). The details of the mechanisms of rotenone-induced MT upregulation in CGA-treated mesencephalic astrocytes and CA/CGA-treated enteric glial cells are unknown. We propose that CA or CGA pretreatment may enhance the reactivity of glial cells to produce antioxidative molecules in response to rotenone exposure.

## 5. Conclusions

Our results demonstrated that coffee components upregulated antioxidative molecules in glial cells and prevented neurodegeneration in the SNpc and the intestinal myenteric plexus in rotenone-treated mice. These results support the epidemiological data that coffee consumption reduces the risk of PD. Our findings indicate that it may be possible to use a food-based promising therapeutic strategy of neuroprotection to improve the motor and non-motor symptoms of PD.
